# High Awareness, Poor Practice: The Cholera Prevention Knowledge‐Attitude‐Practice Gap in Ada Foah, Ghana—A Cross‐Sectional Study

**DOI:** 10.1002/puh2.70339

**Published:** 2026-08-13

**Authors:** Christopher Yaw Dumevi, Princess Afia Takyiwaa Dwomoh, Hugette Naa Ayeley Aryee, Joyce Junior Asiamah, Gifty Selorm Amenya, Gloria Amoo Aidoo, Dorcas Akuokor Teiko, Isaac Ekow Ennin, Eric Essandoh Amuah, James‐Paul Kretchy, Saviour Kweku Adjenti, Patrick F. Ayeh‐Kumi

**Affiliations:** ^1^ Department of Physician Assistantship Studies, School of Medical Sciences Central University Accra Ghana; ^2^ Department of Medical Microbiology University of Ghana Medical School Accra Ghana; ^3^ Department of Public Health, School of Medical Sciences Central University Accra Ghana

**Keywords:** Ada East District, cholera, Ghana, knowledge, attitude, practices

## Abstract

**Background:**

Cholera persists as a public health threat in Ghana, particularly in coastal communities with inadequate water, sanitation and hygiene (WASH) infrastructure. This study assessed the knowledge, attitudes and practices (KAP) toward cholera among residents of Ada Foah, a high‐risk coastal district in Ghana, to identify gaps and inform targeted interventions.

**Methods:**

A community‐based analytical cross‐sectional study was conducted among 400 adults (≥18 years) selected via multi‐stage systematic random sampling. Data were collected using a pretested, structured questionnaire and analysed using descriptive statistics, chi‐square tests, exploratory factor analysis (EFA) and multivariable logistic regression. To address quasi‐complete separation in the practice model, Firth's penalized likelihood regression was applied.

**Results:**

Awareness of cholera was high (93.5%), but significant knowledge gaps persisted regarding aetiology and symptoms. Although attitudes were predominantly positive (80.5% rated as ‘Good’), preventive practices were inconsistent. Notably, 43.5% never treated drinking water, and 40.3% reported practising open defecation ‘sometimes/always’. Higher educational attainment and peri‐urban residence were significant predictors of good knowledge (*p* < 0.05). EFA revealed four distinct practice dimensions: core hygiene, Proactive Food and Water Safety, health system engagement, and risk recognition. Willingness to accept oral cholera vaccine was high (67.0%) despite low awareness (43.0%). Firth's regression confirmed that positive attitude was inversely associated with adequate practice (aOR = 0.04, 95% CI: 0.01–0.18), whereas the four practice factors showed expected directional associations.

**Conclusions:**

A significant attitude–behaviour gap exists, with high awareness and positive attitudes not translating into consistent preventive practices. Cholera control in Ada Foah requires a multifaceted strategy integrating targeted health education, sustainable WASH infrastructure improvements and strategic deployment of oral cholera vaccines.

## Graphical abstract

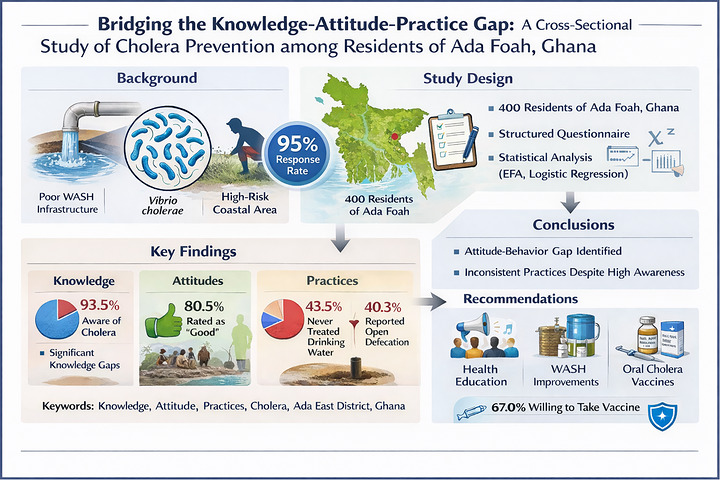

## 1 Introduction

Cholera, an acute secretory diarrhoeal infection caused by the ingestion of the bacterium *Vibrio cholerae* serogroups O1 or O139, remains a potent symbol of inequity and a persistent public health threat in the 21st century [1, 2]. It is estimated that 1.3–4.0 million cases and 21,000–143,000 deaths occur globally each year, with the vast majority of the burden concentrated in regions with inadequate access to safe water, sanitation and hygiene (WASH) facilities [2, 3]. The World Health Organization (WHO) identifies cholera as a key indicator of insufficient social development, and its control is intrinsically linked to the attainment of Sustainable Development Goal (SDG) 6, which aims to ensure availability and sustainable management of water and sanitation for all by 2030 [4].

Cholera spreads primarily through the faecal–oral route via contaminated water and food [5]. The case–fatality rate in well‐managed outbreaks can be below 1%, but this can soar to over 50% in communities with fragile health systems and limited access to care [2, 6]. Effective management relies on rapid rehydration, primarily using oral rehydration solution (ORS), and in severe cases, intravenous fluids and antibiotics [2].

Ghana is situated within the African ‘cholera belt’ and has experienced recurrent outbreaks, as the first case was reported in 1970. The Ghana Health Service reported annual cholera incidence and mortality as follows: 9542 cases with 100 deaths in 2010; 10,628 cases with 105 deaths in 2011; 28,975 cases with 243 deaths in 2014; 618 cases with 5 deaths in 2015; and 150 cases with no reported fatalities in 2016 [7]. Cumulatively, national surveillance data from 1998 to 2017 recorded 82,754 cholera cases, resulting in 519 deaths and an overall case‐fatality ratio of approximately 0.63% [8]. Cholera remains a significant public health threat in Ghana, with recent evidence confirming its persistent burden. A systematic review by Kungu et al. [9] synthesizing data through 2024 reported a pooled cholera prevalence of 18.42% nationally, with a case‐fatality rate of 3.40% and mortality rate of 2.7%. The Greater Accra Region continues as the epicentre, accounting for 58.5% of cases from 1998 to 2017, with recurrent outbreaks driven by suboptimal sanitation and contaminated water sources [10]. These findings underscore the urgent need for integrated control strategies addressing both structural WASH deficiencies and emerging antimicrobial resistance [9].

Despite this documented burden, the success of prevention strategies is contingent upon community‐level knowledge, attitudes and practices (KAP). Understanding these domains is essential for designing effective, culturally appropriate interventions.

The Greater Accra Region, being the most densely populated, is Ghana's persistent cholera epicentre, with Accra Metropolitan Area recording the highest annual incidence. Between 1998 and 2017, the region alone represented 58.5% of the national caseload [10]. The region's rapid, often unplanned urbanization, coupled with perennial flooding and sprawling informal settlements with compromised WASH infrastructure, creates an ideal environment for the propagation of *V. cholerae* [11, 12]. A complex interplay of factors, including open defecation, improper solid waste management and the use of contaminated water sources for domestic purposes, perpetuates the transmission cycle.

The Ada East District, a coastal district in the Greater Accra Region, presents a unique and high‐risk setting for cholera due to its estuarine geography, seasonal flooding and sociocultural practices [13]. Despite these known environmental and structural risk factors, there remains a critical gap in understanding the community's awareness and behavioural disposition toward the disease, which is fundamental for designing targeted interventions.

This study is grounded in the KAP theoretical framework, which posits that improving knowledge shapes attitudes, thereby enabling positive behavioural change [14]. Although the clinical and epidemiological aspects of cholera are well‐documented, the success of prevention strategies ultimately depends on the KAP of the at‐risk population [15, 16]. Studies from other cholera‐endemic regions have consistently shown that poor knowledge of transmission routes, negative attitudes toward preventive measures such as vaccination or chlorination, and high‐risk practices (e.g., consuming untreated water or street food with questionable hygiene) are significant predictors of infection [17, 18]. Understanding these KAP domains is a fundamental prerequisite for designing effective, culturally appropriate and sustainable public health interventions.

In Ghana, few studies have assessed community‐level KAP regarding cholera, and none have specifically focused on the residents of the Ada East District. This gap limits the ability of local health authorities to deploy targeted risk communication and community engagement strategies. Therefore, this study aimed to assess the knowledge, attitudes and preventive practices related to cholera among residents of Ada Foah. The findings are expected to provide an evidence base for tailored public health interventions within a comprehensive cholera control strategy.

## 2 Methods

### 2.1 Study Settings

This study was conducted in Ada Foah, the administrative capital of the Ada East District in the Greater Accra Region of Ghana. The district is a coastal enclave at the estuary of the Volta River, characterized by extensive lagoons and seasonal flooding [13]. This ecology, coupled with reliance on lagoon and river water and challenges in sustainable waste management, creates a persistent high‐risk environment for waterborne diseases like cholera. The area's geography supports livelihoods in fishing and salt production, but sociocultural practices and compromised WASH infrastructure facilitate disease transmission [19]. According to the 2021 Population and Housing Census, the district has a population of 79,411 people, residing in a mix of urban and rural settlements [20]. This specific context establishes Ada Foah as a critical site for investigating the community‐level determinants of cholera prevention.

### 2.2 Study Design and Period

This analytical cross‐sectional study was conducted between 23 June and 30 September 2025. The design is appropriate for estimating the prevalence of KAP components at a single point in time, though it cannot establish causal relationships [21, 22].

### 2.3 Study Participants

The study population included all adult residents (≥18 years) who were permanent inhabitants of selected households in the Ada Foah district, defined as residing in the district for at least 6 months. Eligibility was contingent on the provision of written or thumb‐printed informed consent. Individuals who were severely ill, cognitively unable to participate in an interview or non‐residents were excluded. Although the exclusion of the severely ill was necessary for ethical and practical reasons, we acknowledge this may introduce a minor healthy respondent bias in the sample, potentially leading to a slight overestimation of positive KAP indicators in the community [23].

### 2.4 Sample Size Assumptions and Calculations

The minimum sample size was calculated using Cochran's formula for a single population proportion: *n* = *Z* ^2^ *p*(1 − *p*)/*d* ^2^ [24], where *n* is the sample size; *Z* = 1.96 at a 5% level of significance; *p* = 0.5 is the sample proportion, which provides the maximum sample size; and *d* is the margin of error taken as 5%. Hence, the minimum sample size required for this study was 384. A conservative rate of 10% was assumed to account for potential non‐response, resulting in a final target sample size of 423.

### 2.5 Sampling Strategy

A multi‐stage systematic random sampling technique was employed to select participants, ensuring the sample's representativeness of the Ada Foah population. This probability‐based approach was chosen over non‐probability methods to minimize selection bias and maximize the generalizability of the findings [25].

#### 2.5.1 Sampling Frame and Selection Process

The sampling frame was the official 2022 Household Enumeration List provided by the Ada East District Assembly, constituting a comprehensive and up‐to‐date listing of all residential structures. This verified frame minimized the risk of coverage bias. In the first stage, the district was stratified into peri‐urban and rural areas based on Ghana Statistical Service classifications. In the second stage, households were selected using systematic random sampling within each stratum. The sampling interval (k) was calculated as 15, based on the total number of households and the required sample size (*n* = 404). A random start was selected from the first 15 households, with every 15th household thereafter selected.

In the third stage, within each selected household, a single eligible adult was chosen impartially using the Kish table method [25]. This rigorous, non‐discretionary procedure ensured every eligible member had a known, non‐zero probability of selection, thereby effectively minimizing interviewer selection bias [26]. Survey design effects, including stratification and clustering at the household level, were accounted for in all analyses using survey‐weighted commands in STATA.

#### 2.5.2 Sample Size Considerations and Response Rate

**FIGURE 1:**
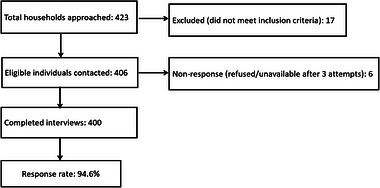
Participant flow diagram.

Of the 423 eligible individuals contacted, 400 completed the interview, yielding a high response rate of 95% (Figure 1). This rate substantially reduces the potential for non‐response bias and strengthens the external validity of the study's findings. The minimum sample size was calculated using Cochran's formula for a single population proportion: *n* = *Z* ^2^ *p*(1 − *p*)/*d* ^2^ [27], where *Z* = 1.96 at a 5% significance level; *p* = 0.5 (proportion providing maximum sample size); and *d* = 0.05 (margin of error). The minimum required sample size was 384, increased by 10% to 423 to account for potential non‐response. The response rate was 95%.

### 2.6 Data Collection

Data were collected using a pretested, structured questionnaire developed after an extensive review of literature on cholera‐related KAP studies [16, 26, 28]. The instrument was meticulously adapted to the local sociocultural and linguistic context to ensure conceptual accuracy and semantic equivalence [29]. The face validity of the study instrument was reviewed by three experts (C.Y.D., J.‐P.K. and P.F.A.‐K.) with backgrounds in medical microbiology, epidemiology and public health. Pretesting with 40 residents from a neighbouring Ningo–Prampram municipality with similar population composition assessed clarity and cultural relevance, leading to minor revisions. Pretest data were excluded from the final analysis.

The questionnaire was organized into four main sections: Socio‐demographic and economic characteristics: age, sex, educational attainment, occupation and household assets; Knowledge of cholera: assessed understanding of cause, transmission routes, clinical symptoms and preventive measures; Attitudes toward cholera: assessed perceived susceptibility, severity and beliefs about the efficacy of preventive and control measures; and Preventive practices: evaluated behaviours related to water treatment, food hygiene, handwashing and safe disposal of faeces and solid waste.

### 2.7 Instrument Development, Pilot Testing and Validation

To ensure validity and reliability, the instrument underwent a multi‐stage process. Content validity was established through review by a panel of three experts (in public health epidemiology, medical microbiology and environmental health), who assessed the relevance, clarity and completeness of items in measuring the target KAP constructs.

#### 2.7.1 Pilot Testing

Face validity and feasibility were assessed via a pilot study conducted in two neighbouring communities with similar characteristics to the study area: Ningo–Prampram (*n* = 20) and Big Ada (*n* = 20), yielding a total of 40 participants. These communities were selected because they share comparable coastal ecology, WASH infrastructure challenges and socio‐demographic profiles with Ada Foah but were excluded from the main study. The pilot tested question comprehension, interview flow and duration. This led to refinements in the wording of ambiguous items and simplification of interview prompts. Pilot data were excluded from the final analysis.

#### 2.7.2 Reliability Assessment

Internal consistency reliability for the multi‐item knowledge and attitude scales was assessed using Cronbach's alpha. The knowledge scale (11 items) demonstrated good reliability (*α* = 0.83), and the attitude scale (9 items) demonstrated acceptable reliability (*α* = 0.77). Construct validity for the preventive practices scale (13 items), which showed poor initial internal consistency (*α* = 0.21), was established using exploratory factor analysis (EFA) with appropriate reverse‐coding of risk behaviours as described below.

#### 2.7.3 Bias Mitigation

To mitigate social desirability bias particularly for sensitive behaviours such as open defecation and water treatment, research assistants underwent intensive 2‐day training focused on neutral questioning techniques. Interviewers were instructed to: (1) preface sensitive questions with normalizing statements (e.g., ‘In many communities, people face challenges with…’); (2) use non‐judgmental tone and body language; (3) emphasize confidentiality and the absence of right or wrong answers; and (4) allow participants to respond in their own words before coding. Regular field supervision and daily debriefing sessions reinforced these techniques throughout data collection.

### 2.8 Psychometric Evaluation of the Practices Scale

Cholera prevention practices were assessed using 13 items. Prior to analysis, all risk behaviour items (practising open defecation, eating street food prepared in unhygienic conditions and consuming potentially contaminated food) were reverse‐coded so that higher scores consistently represented safer practices across all items. Initial validation of a unidimensional scale combining all items revealed unacceptably low internal consistency (Cronbach's *α* = 0.21 prior to reverse‐coding), with several items exhibiting negative item‐total correlations prior to reverse‐coding, indicating multidimensionality and inconsistent scoring direction. After reverse‐coding, item‐total correlations improved but remained suboptimal for a single composite measure.

Therefore, we employed EFA with principal component analysis extraction and Varimax rotation to identify underlying constructs. The Kaiser–Meyer–Olkin (KMO) measure of sampling adequacy was 0.72, and Bartlett's test of sphericity was significant (*p* < 0.001), confirming the suitability of the data for factor analysis. The derived factors were then assessed for internal consistency.

The number of practice items was consistently 13 throughout the analysis; any perceived inconsistency in reporting reflects the transition from the full 13‐item set to the final factor‐derived subscales, each comprising different subsets of these items.

### 2.9 Scoring of KAP

For the knowledge section, correct responses were assigned a score of 1, whereas incorrect or ‘unsure’ responses were assigned a score of 0. The total knowledge score was converted to a percentage. For the primary analysis using multivariable logistic regression, knowledge level was categorized dichotomously as ‘good knowledge’ (≥70%) or ‘poor knowledge’ (<70%). For descriptive analyses and to explore distributions in greater detail, a three‐category classification was also utilized: ‘Poor’ (<50%), ‘Moderate’ (50%–69%) and ‘Good’ (≥70%).

Attitude statements were measured on a three‐point Likert scale (Agree, Disagree and Unsure). Positive attitude statements were assigned a score of 1 for ‘Agree’ and 0 for ‘Disagree’ or ‘Unsure’; negative attitude statements were reverse‐coded accordingly. A composite attitude score was calculated as a percentage and classified as ‘positive attitude’ (≥70%) or ‘negative attitude’ (<70%).

#### 2.9.1 Justification for Threshold Selection

The 70% threshold for knowledge and attitude dichotomization was selected on the basis of Bloom's cut‐off criteria, which is widely used in KAP studies to denote adequate mastery of health information [30, 31]. For practices, an 80% threshold was applied to reflect the higher standard required for protective behaviours that directly impact disease transmission. This higher benchmark aligns with competency‐based assessments in preventive health, where consistent adherence rather than occasional compliance is necessary to interrupt faecal–oral transmission routes [32, 33]. The 80% threshold therefore accounts for the critical nature of WASH practices in cholera prevention.

Practice items were scored on the basis of self‐reported frequency of adherence to recommended preventive behaviours. Risk behaviours (e.g., open defecation, consuming potentially contaminated food) were reverse‐coded so that higher scores consistently indicated safer practices. A composite practice score was calculated, and participants were classified as having ‘adequate practice’ if they reported adherence to ≥80% of key preventive behaviours. The use of self‐reported data for practices is acknowledged as a potential source of social desirability bias; this was mitigated through rigorous interviewer training as described above.

### 2.10 Data Analysis

Data were entered into Microsoft Excel, cleaned, and analysed using IBM SPSS Statistics Version 26.0, with STATA version 17.0 used for advanced regression modelling and robustness checks. All analyses incorporated survey weights to account for the complex sampling design, including stratification by residential area (peri‐urban vs. rural) and clustering at the household level. These survey‐adjustment procedures were implemented using survey‐specific commands in STATA to produce appropriate standard errors and population‐representative estimates.

Descriptive statistics were computed to summarize the characteristics of the study population and the KAP outcomes. Continuous variables were presented as means (standard deviation) or medians (interquartile range), whereas categorical variables were summarized as frequencies and percentages. The primary dependent variables were knowledge level (good/poor), attitude (positive/negative) and practice (adequate/inadequate).

Bivariate analyses, utilizing chi‐square tests (or Fisher's exact test where appropriate) for categorical variables and independent samples *t*‐tests for continuous variables, were conducted to identify candidate variables associated with the outcomes. All independent variables with a *p* value < 0.20 in bivariate analysis were considered for inclusion in the multivariable logistic regression models. Model building followed a combination of statistical significance and theoretical importance, with established confounders (age, education and wealth) retained in final models regardless of bivariate *p* value.

#### 2.10.1 Handling of Quasi‐Complete Separation

Initial multivariable logistic regression for the ‘good practice’ outcome revealed evidence of quasi‐complete separation, characterized by extremely wide confidence intervals and implausibly large odds ratios (e.g., aOR = 488.12 for Risk Recognition and Vaccine Intent). This statistical phenomenon occurs when a predictor perfectly or near‐perfectly predicts the outcome, leading to unstable maximum likelihood estimates. To address this, we applied Firth's penalized likelihood logistic regression [33], a bias‐reduction method designed specifically for separation issues in logistic regression. Firth's regression introduces a small penalty to the likelihood function, producing finite, consistent estimates even with complete or quasi‐complete separation. Results from Firth's regression are presented alongside conventional estimates for comparison, with primary interpretation based on the penalized models.

The dependent variables were defined as follows: knowledge (factual understanding of cholera's cause, transmission, symptoms and prevention), attitude (subjective beliefs, perceived risk, and outlook toward cholera and its control measures) and practices (self‐reported actions related to cholera prevention). The independent variables included a range of socio‐demographic and economic characteristics.

Additionally, although we constructed a multivariable logistic regression model for ‘good practice’, the initial conventional estimates exhibited separation. Consequently, we present Firth's penalized regression results as the primary analytic output for this outcome. Due to the demonstrated multidimensionality of practices, we also emphasize interpretation of the derived practice factors from the EFA as complementary, nuanced behavioural outcomes.

## 3 Results

A total of 450 households were approached, of which 404 met eligibility criteria and were invited to participate. Among these, 400 individuals completed the interview, yielding a response rate of 95% (Figure 1). Four eligible individuals declined participation or were unavailable after three contact attempts. All 400 completed interviews were included in the final analysis.

### 3.1 Socio‐Demographic Characteristics of the Study Participants

The study comprised 400 participants with the gender distribution nearly equivalent, with 213 females (53.3%) and 187 males (46.8%). The mean age of participants was 27.25 years (SD = 10.23). The majority (57.5%, *n* = 230) were aged 20–29 years, with 15.0% (*n* = 60) under 20 years, indicating a predominantly young adult sample. Regarding marital status, most participants were single (68.8%, *n* = 275). Christians constituted the largest religious group (72.8%, *n* = 291). More than half of the participants resided in peri‐urban areas (58.8%, *n* = 235).

Educational attainment varied, with the largest proportion having completed secondary education (37.3%, *n* = 149), followed by tertiary education (29.5%, *n* = 118). A notable proportion (11.8%, *n* = 47) reported no formal education. Occupationally, students represented the largest single group (27.8%, *n* = 111), followed by government employees (24.5%, *n* = 98) and private sector employees (24.3%, *n* = 97) (Table 1).

**TABLE 1:** Socio‐demographic characteristics of the study participants.

Variable	Category	*n* (%)
Gender	Male	187 (46.8)
	Female	213 (53.3)
Age	Mean ± SD	27.25 ± 10.23
Age grouped	Less than 20	60 (15.0)
	20–29	230 (57.5)
	30–39	53 (13.3)
	40–49	40 (10.0)
	50–59	10 (2.5)
	60 and above	7 (1.8)
Marital status	Single	275 (68.8)
	Married	83 (20.8)
	Divorced	14 (3.5)
	Widowed	8 (2.0)
	Cohabiting	20 (5.0)
Religion	Christian	291 (72.8)
	Muslim	82 (20.5)
	Traditionalist	27 (6.8)
Residence	Peri‐urban	235 (58.8)
	Rural	165 (41.3)
Education	No formal education	47 (11.8)
	Basic school	86 (21.5)
	Secondary	149 (37.3)
	Tertiary	118 (29.5)
Occupation	Unemployed	35 (8.8)
	Fishing	15 (3.8)
	Farmer	44 (11.0)
	Government employee	98 (24.5)
	Private sector employee	97 (24.3)
	Student	111 (27.8)

### 3.2 Household Water and Food Safety Practices

**FIGURE 2:**
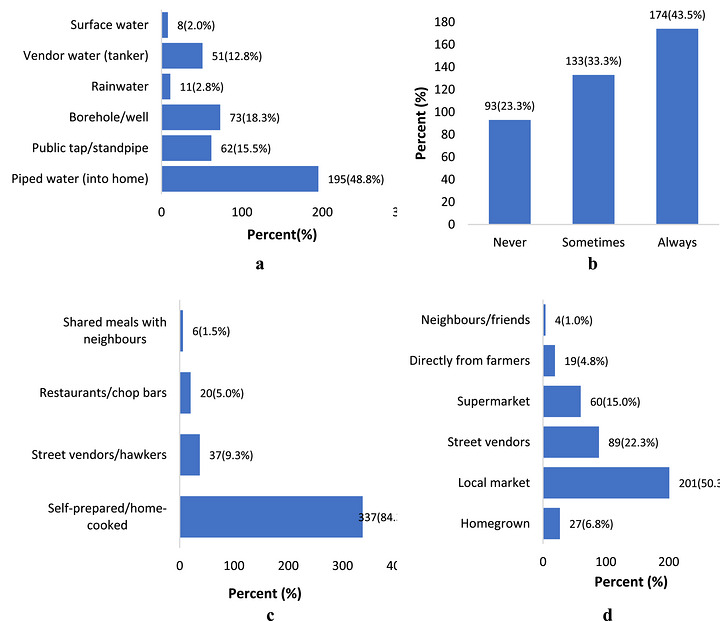
(a) Household's primary drinking water source. (b) Water treatment before drinking. (c) Main source of food. (d) Main source of fruits/vegetables.

Household water and food safety practices are summarized in Figure 2a,d. Notably, although piped water was the primary drinking source for 48.8% of households, 43.5% reported never treating their water before consumption.

### 3.3 Awareness and Source of Information About Cholera

**FIGURE 3:**
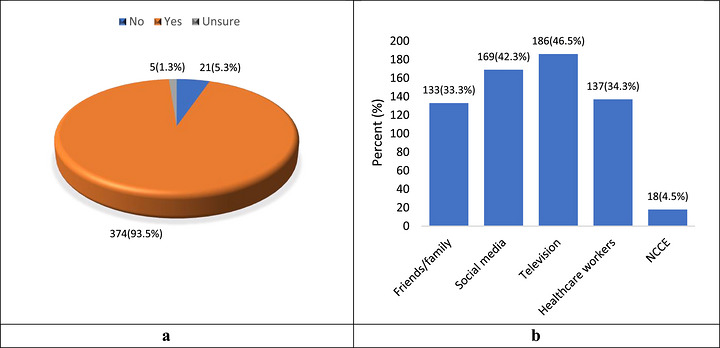
(a) Have you heard of cholera? (b) Source of information about cholera.

Nearly all respondents (93.5%) had heard of cholera, with information primarily sourced from friends/family (46.5%) and social media (42.3%), followed by television (34.3%) as shown in Figure 3a,b.

### 3.4 Knowledge of Cholera Aetiology, Transmission, Symptoms and Key Facts

**FIGURE 4:**
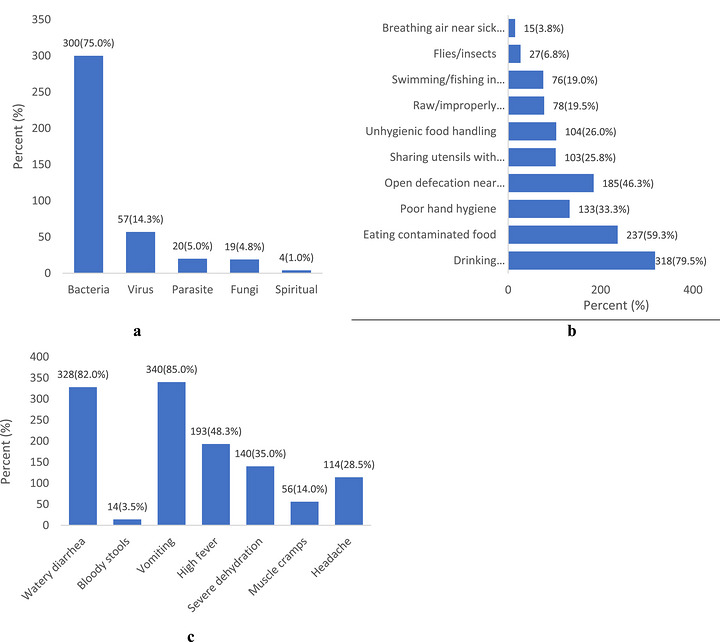
(a) Aetiology of cholera. (b) Possible ways to contract cholera. (c) Symptoms of cholera.

As shown in Figure 4a,c and Table 2, most respondents correctly identified bacterial aetiology (75.0%) and contaminated water as a transmission route (79.5%). However, notable gaps persisted: only 46.3% recognized open defecation as a risk, and less than half (48.3%) knew that watery diarrhoea is a symptom.

**TABLE 2:** Knowledge of prevention, treatment and epidemiology.

Other knowledge items	Yes	No	Unsure
Can cholera be prevented?	357 (89.3)		43 (10.8)
Does poor personal hygiene increase cholera risk?	360 (90.0)		40 (10.0)
Is cholera treatable?	363 (90.8)	4 (1.0)	33 (8.3)
Can a person get cholera again after recovery?	331 (82.8)	23 (5.8)	46 (11.5)
Does eating/drinking contaminated food/water increase cholera risk?	351 (87.8)	4 (1.0)	45 (11.3)
Do infected individuals always show symptoms?	346 (86.5)	9 (2.3)	45 (11.3)
Does handwashing with soap prevent cholera?	353 (88.3)	9 (2.3)	38 (9.5)

### 3.5 Knowledge of Aetiology, Transmission and Symptoms

### 3.6 Association of Cholera Knowledge With Respondent Demographics

Table 3 presents the bivariate associations between demographic characteristics and cholera knowledge categories. Educational attainment and age group were the only variables significantly associated with knowledge level (*p* < 0.001 for both), with higher education and younger age (20–49 years) concentrated in the ‘good’ knowledge category.

**TABLE 3:** Association of knowledge and demographics of respondents.

Variable	Category	Knowledge category			Total	*p* value	95% CI
		Poor	Moderate	Good			
Sex	Male	123 (49.8)	57 (42.5)	7 (36.8)	187 (46.8)	0.269	0.229–0.316
	Female	124 (50.2)	77 (57.5)	12 (63.2)	213 (53.3)		
Age group	Less than 20	50 (20.2)	10 (7.5)	0 (0.0)	60 (15.0)	<0.001	0.000–0.007
	20–29	135 (54.7)	83 (61.9)	12 (63.2)	230 (57.5)		
	30–39	26 (10.5)	27 (20.1)	0 (0.0)	53 (13.3)		
	40–49	24 (9.7)	9 (6.7)	7 (36.8)	40 (10.0)		
	50–59	10 (4.0)	0 (0.0)	0 (0.0)	10 (2.5)		
	60 and above	2 (0.8)	5 (3.7)	0 (0.0)	7 (1.8)		
Marital status	Single	175 (70.9)	90 (67.2)	10 (52.6)	275 (68.8)	0.274	0.205–0.290
	Married	47 (19.0)	30 (22.4)	6 (31.6)	83 (20.8)		
	Divorced	10 (4.0)	4 (3.0)	0 (0.0)	14 (3.5)		
	Widowed	6 (2.4)	2 (1.5)	0 (0.0)	8 (2.0)		
	Cohabiting	9 (3.6)	8 (6.0)	3 (15.8)	20 (5.0)		
Residential area	Peri‐urban	137 (55.5)	85 (63.4)	13 (68.4)	235 (58.8)	0.218	0.198–0.282
	Rural	110 (44.5)	49 (36.6)	6 (31.6)	165 (41.3)		
Highest educational level	No formal education	42 (17.0)	4 (3.0)	1 (5.3)	47 (11.8)	<0.001	0.000–0.007
	Basic school	70 (28.3)	14 (10.4)	2 (10.5)	86 (21.5)		
	Secondary	76 (30.8)	59 (44.0)	14 (73.7)	149 (37.3)		
	Tertiary	59 (23.9)	57 (42.5)	2 (10.5)	118 (29.5)		

*Note:* Three‐category classification used for descriptive analysis. For regression analysis (Table 9), knowledge was modelled dichotomously (good [≥70%] vs. poor [<70%]).

### 3.7 Attitudes Toward Cholera Prevention

Attitudes toward cholera prevention were positive across most items (Table 4), with over 85% of respondents affirming the importance of personal hygiene, handwashing and medical care. A notable exception was that 40.0% felt uncomfortable reminding others to wash hands.

**TABLE 4:** Attitudes toward cholera prevention.

	Response		
Attitude item	No	Yes	Unsure
Is cholera a serious concern in your community?	87 (21.8)	252 (63.0)	61 (15.3)
Can cholera lead to death?	21 (5.3)	315 (78.8)	64 (16.0)
Are you willing to take steps to protect your family from cholera?	4 (1.0)	378 (94.5)	18 (4.5)
Does washing vegetables reduce cholera risk?	26 (6.5)	344 (86.0)	30 (7.5)
Do you feel uncomfortable reminding others to wash hands?	202 (50.5)	160 (40.0)	38 (9.5)
Is personal hygiene critical for cholera prevention?	30 (7.5)	335 (83.5)	35 (8.8)
Does proper faecal disposal prevent cholera?	72 (18.0)	277 (69.3)	51 (12.8)
Is seeking medical care important for diarrhoea/vomiting?	15 (3.8)	357 (89.3)	28 (7.0)
Is buying food from dirty vendors risky?	17 (4.3)	341 (85.3)	42 (10.5)

### 3.8 Distribution of Attitude Categories Among Respondents

**FIGURE 5:**
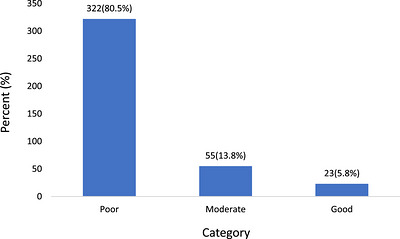
Distribution of attitude categories among respondents.

The overall attitude of respondents toward cholera prevention was predominantly positive. As shown in Figure 5, the majority of respondents (80.5%, *n* = 322) were categorized as having a ‘Good’ attitude. A smaller proportion exhibited a ‘Poor’ attitude (13.8%, *n* = 55), whereas the ‘Moderate’ category contained the fewest respondents (5.8%, *n* = 23).

### 3.9 Practices Related to Cholera Prevention

Self‐reported practices varied considerably by domain (Tables 5a and 5b). Although hand hygiene adherence was relatively high (>70% always), critical gaps were observed in water treatment (43.5% never treat) and open defecation (40.3% practice sometimes/always). Vaccine willingness (67.0%) far exceeded awareness (43.0%).

**TABLE 5a:** Practices related to cholera prevention.

Other knowledge items	Never	Sometimes	Always
Do you wash hands with soap after using the toilet?	3 (0.8)	107 (26.8)	290 (72.5)
Do you wash hands before eating/preparing food?	0 (0.0)	120 (30.0)	280 (70.0)
Do you cover food to prevent flies?	15 (3.8)	125 (31.30	260 (65.0)
Do you avoid unwashed fruits/vegetables?	44 (11.0)	155 (38.8)	201 (50.2)
Do you eat street food prepared in unhygienic conditions?	187 (46.8)	139 (34.8)	74 (18.5)
Do you store drinking water in a covered container?	52 (13.0)	129 (32.3)	219 (54.8)
Do you practice open defecation?	239 (59.8)	111 (27.8)	50 (12.5)
Do you buy food from hygienic sources?	12 (3.0)	114 (28.5)	274 (68.5)

**TABLE 5b:** Practices related to cholera prevention.

Other knowledge items	Yes	No	Unsure
Have you eaten potentially contaminated food?	100 (25.0)	171 (42.8)	129 (32.3)
Have you attended any cholera health education session?	190 (47.5)	192 (48.0)	18 (4.5)
Are you aware of cholera vaccines?	172 (43.0)	180 (45.0)	48 (12.0)
Would you take a cholera vaccine if available?	268 (67.0)	98 (24.5)	34 (8.5)

### 3.10 Psychometric Evaluation of the Practices Scale

Initial validation indicated that preventive practices constituted a multidimensional construct. EFA was therefore conducted, yielding a robust four‐factor solution (see S2 Table 6) which accounted for 58.42% of the total variance. These reliably measured factors were used as predictor variables in subsequent regression models.

Initial psychometric evaluation of a 10‐item composite practices scale indicated very poor internal consistency (*α* = 0.211). Item‐total statistics confirmed fundamental scale incompatibility, with items measuring risk behaviours (e.g., practising open defecation; *r* = −0.268) correlating negatively with items measuring preventive behaviours (e.g., handwashing with soap; *r* = 0.381). Consequently, EFA was conducted, yielding a robust four‐factor solution (see S2 Table 6). The internal consistency of these derived multi‐item factors was excellent: Proactive Food and Water Safety (*α* = 0.913), Risk Recognition and Vaccine Intent (*α* = 0.862), Risk–Prevention Behaviour (*α* = 0.799) and Health System Engagement (*α* = 0.754). The vaccine acceptance item loaded appropriately on its factor and demonstrated a positive item‐total correlation (*r* = 0.210), whereas general vaccine awareness was a weaker indicator (Table 6).

**TABLE 6:** Psychometric evaluation of the practices scale.

	Scale mean if item deleted	Scale variance if item deleted	Corrected item‐total correlation	Squared multiple correlation	Cronbach's alpha if item deleted
Do you wash hands with soap after using the toilet?	18.9925	5.220	0.381	0.563	0.111
Do you wash hands before eating/preparing food?	19.0100	5.343	0.329	0.630	0.132
Have you eaten potentially contaminated food?	20.8150	5.815	−0.073	0.099	0.311
Do you cover food to prevent flies?	19.0975	5.391	0.210	0.555	0.162
Do you avoid unwashed fruits/vegetables?	19.3175	5.155	0.207	0.196	0.150
Do you eat street food prepared in unhygienic conditions?	19.9925	6.489	−0.210	0.203	0.365
Do you store drinking water in a covered container?	19.2925	5.466	0.085	0.105	0.213
Do you practice open defecation?	20.1825	6.736	−0.268	0.201	0.380
Do you buy food from hygienic sources?	19.0550	5.691	0.107	0.228	0.206
Have you attended any cholera health education session?	21.1450	5.282	0.237	0.161	0.147
Are you aware of cholera vaccines?	21.0400	5.432	0.113	0.140	0.199
Would you take a cholera vaccine if available?	20.8700	5.407	0.210	0.119	0.163

### 3.11 Factor Structure of Cholera Prevention Practices

Principal component analysis with Varimax rotation yielded a four‐component solution accounting for 58.42% of the total variance in the 10 practice items’ KMO value (0.72); Bartlett's test *p* < 0.001). Component 1 (Core Hygiene and Food Protection; 28.22% variance) was defined by high loadings from handwashing before meals (0.866), handwashing after toilet use (0.837), and covering food from flies (0.819). Component 2 (Proactive Food and Water Safety; 12.73%) comprised storing water in a covered container (0.707) and avoiding unwashed produce (0.532). Component 3 (Health System Engagement; 9.07%) included attendance at health education (0.800) and vaccine awareness (0.770). Component 4 (Risk Recognition and Vaccine Intent; 8.40%) linked self‐reported consumption of contaminated food (0.766) with willingness to accept a cholera vaccine (0.634). The internal consistency of the resulting multi‐item factors was assessed. The derived scales demonstrated good to excellent reliability: Proactive Food and Water Safety (three items, *α* = 0.913), Risk Recognition and Vaccine Intent (two items, *α* = 0.862), Risk–Prevention Behaviour (four items, *α* = 0.799) and Health System Engagement (two items, *α* = 0.754) (Table 7, 8, 9). These reliably measured factors were subsequently used as predictor variables in regression models.

**TABLE 7:** Rotated component matrix.

	Component			
	1	2	3	4
Do you wash hands before eating/preparing food?	0.866			
Do you wash hands with soap after using the toilet?	0.837			
Do you cover food to prevent flies?	0.819			
Do you buy food from hygienic sources?	0.426			
Do you avoid unwashed fruits/vegetables?		0.532		
Do you store drinking water in a covered container?		0.707		
Have you attended any cholera health education session?			0.800	
Are you aware of cholera vaccines?			0.770	
Have you eaten potentially contaminated food?				0.766
Would you take a cholera vaccine if available?				0.634
% variance explained	28.22	12.73	9.07	8.40
Cumulative % variance explained	28.22	40.95	50.02	58.42
Extraction method: principal component analysis Rotation method: varimax with Kaiser normalization					

**TABLE 8:** Reliability of extracted factors.

Scale	Mean ± SD	Cronbach's alpha
Core Hygiene and Food Protection	10.69 ± 1.60	0.799
Proactive Food and Water Safety	4.81 ± 1.07	0.913
Health System Engagement	1.24 ± 1.02	0.754
Risk Recognition and Vaccine Intent	1.74 ± 1.09	0.862

**TABLE 9:** Survey‐adjusted and Firth's penalized logistic regression predicting good knowledge, positive attitude and adequate practice.

Predictors	Knowledge (≥70%) aOR (95% CI)	Attitude (≥70%) aOR (95% CI)	Practice (≥80%) Conventional aOR (95% CI)	Practice (≥80%) Firth's aOR (95% CI)
Age (years)	1.02 (0.98–1.06)	1.05 (1.01–1.10)*	1.00 (0.81–1.25)	1.01 (0.97–1.05)
Sex (male vs. female)	0.72 (0.42–1.22)	1.49 (0.76–2.98)	3.62 (0.35–37.35)	1.28 (0.68–2.41)
Education (ref: no formal)				
Basic school	4.00 (0.94–17.02)	1.00 (0.34–3.30)	9.80 (0.58–167.53)	2.18 (0.71–6.69)
Secondary	13.40 (3.19–56.35)**	2.47 (0.72–8.54)	0.04 (0.01–0.98)*	0.42 (0.14–1.27)
Tertiary	11.94 (2.84–50.11)**	0.80 (0.22–2.96)	1.71 (1.38–21.11)*	1.93 (0.66–5.63)
Residence (peri‐urban vs. rural)	1.90 (1.07–3.40)*	1.04 (0.52–2.10)	0.55 (0.80–0.98)*	0.81 (0.42–1.56)
Attitude (positive vs. negative)	—	—	0.00 (0.00–0.01)**	0.04 (0.01–0.18)**
Practice factors				
Core Hygiene and Food Protection	1.16 (0.96–1.40)	0.66 (0.53–0.82)**	14.93 (4.48–49.81)**	2.84 (1.62–4.98)**
Proactive Food/Water Safety	1.05 (0.84–1.33)	0.62 (0.42–0.92)*	48.46 (4.28–549.41)**	3.17 (1.41–7.12)**
Health System Engagement	1.54 (1.20–1.97)**	0.54 (0.38–0.77)**	114.68 (2.96–4446.39)*	2.93 (1.18–7.28)*
Risk Recognition/Vaccine Intent	1.46 (1.13–1.89)**	2.49 (1.66–3.74)**	488.12 (16.80–14,182.88)**	3.89 (1.54–9.82)**

*Note:* All models adjusted for marital status, occupation, and food source. Conventional practice model exhibited quasi‐complete separation; Firth's penalized likelihood regression provides more stable estimates.

**p < 0.01.

*p < 0.05.

For use in subsequent regression analyses (Table 9), these four components were labelled as follows for clarity and consistency: Component 1 (Core Hygiene and Food Protection); Component 2 (Proactive Food and Water Safety); Component 3 (Health System Engagement) and Component 4 (Risk Recognition and Vaccine Intent) were termed ‘Risk Recognition and Vaccine Intent’.

Prior to factor analysis, all risk behaviour items (practising open defecation, eating street food prepared in unhygienic conditions and consuming potentially contaminated food) were reverse‐coded so that higher scores consistently represented safer practices across all items. The four derived factors were not pre‐specified but were empirically identified through EFA. Each factor therefore represents a distinct behavioural domain: *Core Hygiene and Food Protection* (handwashing, covering food), *Proactive Food and Water Safety* (water storage, avoiding unwashed produce), *Health System Engagement* (attendance at education sessions, vaccine awareness) and *Risk Recognition and Vaccine Intent* (acknowledgment of exposure, vaccine willingness). This empirical grouping enhances transparency by linking analytical categories directly to observed data patterns rather than a priori assumptions.

The reliability analysis yielded the following results (see S3 Table 8). The Core Hygiene and Food Protection scale had a mean score of 10.69 (SD ± 1.60) with a Cronbach's alpha of 0.799; the Proactive Food and Water Safety scale showed a mean of 4.81 (SD ± 1.07) and an alpha of 0.913; the Health System Engagement scale had a mean of 1.24 (SD ± 1.02) and an alpha of 0.754; and the Risk Recognition and Vaccine Intent scale demonstrated a mean of 1.74 (SD ± 1.09) with an alpha of 0.862 (Table 8).

### 3.12 Factors Associated With KAP Outcomes

Table 9 presents the multivariable regression results for good knowledge, positive attitude and adequate practice. For the practice outcome, conventional logistic regression exhibited quasi‐complete separation; therefore, Firth's penalized likelihood estimates are presented alongside conventional estimates for comparison.

## 4 Discussion

This study assessed the cholera‐related KAP among residents of Ada Foah, Ghana. The central finding is a noticeable attitude–behaviour gap: Although awareness (93.5%) and positive attitudes (80.5%) were high, this did not translate into consistent preventive practices. Specifically, nearly half of households, despite being aware of the importance of water treatment, reported never treating water from a likely contaminated source before consumption.

The finding that household drinking water quality poses a significant risk is consistent with previous research in the region. A community‐based cross‐sectional study conducted in both cholera‐endemic and non‐endemic areas of the Greater Accra Region of Ghana revealed highly contaminated household drinking water with coliform bacteria, which poses a serious risk for waterborne diseases like cholera [34]. This finding emphasizes the need for effective water treatment strategies to mitigate the potential negative impact of consuming contaminated water.

The potential for effective intervention is supported by evidence from other settings. In response to a cholera outbreak in Haiti, a public health intervention focused on high‐risk communities, such as resource‐limited neighbourhoods of Port‐au‐Prince, demonstrated a significant increase in self‐reported household water treatment practices, rising from 30.3% to 73.9% [35]. This outcome demonstrates the positive effect of induced behaviour change achieved through a targeted public health strategy.

A significant knowledge deficit was identified, with more than half of the study respondents unaware that open defecation poses a serious risk for cholera transmission. This gap in understanding is detrimental to effective cholera control and prevention strategies. This risk is exacerbated in many coastal communities where WASH infrastructure, such as covered pit latrines for low‐income households, is either limited or entirely absent. This infrastructural deficit compels residents to engage in open defecation and maintain suboptimal hygiene practices. This local challenge aligns with national data, which indicates that open defecation is a major contributor to cholera outbreaks in Ghana, with approximately 19% of the population engaging in this practice [36, 37]. Open defecation facilitates the faecal‐oral transmission route, directly linking inadequate sanitation to the burden of waterborne diseases.

The findings from our study are consistent with global literature emphasizing the critical role of open defecation in cholera transmission. For instance, open defecation is widely acknowledged as a significant risk factor for cholera in Kenya [38]. Furthermore, modelling studies by Olaiju et al. [39] in Nigeria suggest that optimal cholera control measures targeting open defecation could reduce peak infection by 60% and decrease the practice of open defecation by 40%. Conversely, the persistence of behavioural challenges is highlighted in a study conducted in India by Deoshatwar [40], who reported that approximately 62.8% of people in India continued to practice open defecation even in areas with access to toilet facilities.

Awareness of cholera as a public health threat was near‐universal. However, significant gaps in specific knowledge persisted, particularly regarding aetiology and symptoms. The persistence of spiritual causal beliefs (25.5%) underscores the reality of medical pluralism in many communities, where biomedical and traditional/religious health paradigms operate concurrently [41, 42]. Such beliefs do not necessarily indicate a rejection of biomedical information but may represent a complementary framework for understanding causation, particularly for sudden, severe or stigmatized outbreaks like cholera. Public health strategies that dismiss these beliefs risk alienating community segments. Conversely, approaches that recognize pluralism can tailor messaging to address spiritual concerns while effectively conveying actionable biomedical prevention steps [43, 44].

Knowledge was strongly and independently associated with formal education and peri‐urban residence. Respondents with secondary or tertiary education had 11.9–13.4 times higher odds of good knowledge than those with no formal education, reinforcing education's foundational role in health literacy. The concentration of good knowledge among adults under 50 and its absence among the very young and elderly identifies programmatically vulnerable subgroups. This stratification provides a clear map for targeting health education campaigns to those most at risk and least reached by current information channels.

Attitudes toward cholera prevention were overwhelmingly positive, with strong consensus on disease severity and the value of preventive measures like hygiene and medical care. This community‐wide positive disposition is a substantial asset for intervention. However, two nuanced findings warrant attention. First, 40% reported discomfort in reminding others about hand hygiene, suggesting a barrier to the peer reinforcement crucial for sustaining community‐wide norms.

Second, regression analysis revealed married individuals had significantly lower odds of a positive attitude than their single counterparts (aOR = 0.19). This may be interpreted through the lens of perceived behavioural control, a core construct of the health belief model and theory of planned behaviour [45, 46]. Married individuals, who often bear primary responsibility for household water, sanitation and health, may have a more acute and realistic awareness of structural constraints (e.g., the recurring cost of water treatment and the absence of latrines). This daily confrontation with barriers can diminish perceived self‐efficacy and outcome efficacy regarding cholera prevention, leading to a more constrained or fatalistic outlook compared to single individuals, who may perceive preventive behaviours as more personally achievable [47, 48]. These findings align with recent calls for integrated cholera control strategies that move beyond individual‐level interventions. As Khan and Kamil [49] argue, the persistent cholera crisis requires comprehensive approaches combining epidemiological surveillance, WASH infrastructure development and community engagement—a framework that directly supports the multifaceted strategy recommended for Ada Foah.

The attitude–behaviour gap observed in this study warrants careful interpretation within the context of cross‐sectional design. Although we document a significant discrepancy between positive attitudes and consistent preventive practices, this snapshot cannot establish the temporal process through which knowledge and attitudes translate (or fail to translate) into behaviour over time. Longitudinal studies would be valuable to track how individuals navigate barriers to practice adoption, particularly as WASH infrastructure improves or health education campaigns intensify. Nevertheless, the magnitude of the gap, with 80.5% holding positive attitudes but only 43.5% consistently treating water, suggests that barriers are substantial and systemic rather than transient.

A key methodological insight of this study is the empirical demonstration that cholera prevention practices are not unidimensional but consist of four distinct behavioural domains, as revealed by EFA: (1) Core Hygiene and Food Protection, (2) Proactive Food and Water Safety, (3) Health System Engagement and (4) Risk Recognition and Vaccine Intent. This multidimensionality clarifies the attitude–behaviour gap: Translation from intention to action depends on the specific practice domain and its unique barriers.

For instance, *Core Hygiene and Food Protection* (e.g., handwashing and covering food) showed relatively high adherence, likely reflecting the success of long‐standing individual‐focused hygiene campaigns. In stark contrast, practices under *Proactive Food and Water Safety* (e.g., treating water and avoiding unwashed produce) showed critical deficits. This critical divergence underscores that the KAP gap is not primarily a failure of individual motivation for some behaviours, but a direct consequence of inadequate WASH infrastructure that makes safe practice physically or economically impossible. The attitude–behaviour gap in Ada Foah is inextricably linked to its environmental and infrastructural context. As a flood‐prone coastal district, the ecology facilitates the faecal–oral transmission cycle. Therefore, bridging this gap requires moving beyond individual‐level education to address systemic failures through a One Health approach that integrates human behaviour change with environmental management.

Notably, findings related to the *Health System Engagement* and *Risk Recognition and Vaccine Intent* domains reveal a critical programmatic opportunity. Willingness to accept a cholera vaccine was high (67.0%) despite low awareness of its existence (43.0%). This suggests the community presents low vaccine hesitancy but a significant information gap. The programmatic challenge thus shifts from combating resistance to one of demand generation and reliable supply, a fundamentally different and potentially more straightforward operational task for the health system.

### 4.1 Study Limitations and Strengths

The interpretations of this study must be considered alongside its limitations. First, the cross‐sectional design precludes causal inference and cannot establish the temporal sequence through which knowledge and attitudes influence practices. Second, self‐reported practices are susceptible to social desirability bias, potentially leading to over‐reporting of positive behaviours like handwashing. However, we implemented rigorous interviewer training focused on neutral questioning techniques and used normalizing statements for sensitive behaviours (e.g., open defecation) to minimize this bias. Third, the exclusion of severely ill individuals may have introduced a minor healthy‐respondent bias, potentially leading to slight overestimation of positive KAP indicators. Fourth, the practice scale demonstrated moderate internal consistency, and although Firth's regression addressed separation issues, some imprecision in estimates remains. Fifth, data collection occurred during a specific period (June–September 2025), and findings may not capture seasonal variations in knowledge or practices.

Additionally, although Firth's penalized likelihood regression provided stable estimates in the presence of quasi‐complete separation, the separation itself reflects the strong predictive relationship between certain practice factors and the outcome. Consequently, the magnitude of odds ratios should be interpreted as indicating the strength of these associations rather than as precise population estimates.

However, the study's strengths are considerable: a robust, multi‐stage sampling strategy with clear documentation of sampling frame and selection procedures; a very high response rate (95%); the use of validated and locally adapted instruments with rigorous translation and pilot testing; advanced analytical methods, including EFA to identify multidimensional practice domains; and application of Firth's penalized regression to address statistical separation. These strengths enhance the validity and generalizability of the findings within the Ada East District.

## 5 Conclusion and Recommendation

In conclusion, cholera prevention in Ada Foah is hindered by a complex interplay of specific knowledge gaps among vulnerable groups, structural WASH deficiencies that make safe practices impractical, and a demonstrated failure to convert positive attitudes into action where structural barriers are highest. The multidimensional nature of practices, as empirically defined in this study, underscores that interventions must be equally multifaceted and targeted. Effective control requires an integrated strategy that simultaneously: (1) targets mindsets through demographically tailored education correcting specific misconceptions; (2) changes the environment through sustained investment in water treatment and sanitation infrastructure; (3) leverages the health system by capitalizing on high vaccine acceptability through strategic oral cholera vaccine deployment and awareness campaigns and (4) strengthens community norms by addressing social barriers to peer promotion of hygiene. This multi‐pronged approach is essential to disrupt the transmission cycle and sustainably bridge the critical KAP gap identified in this high‐risk community.

## Author Contributions

Christopher Yaw Dumevi conceived and designed the study. Princess Afia Takyiwaa Dwomoh participated in the field investigations and data collection. Christopher Yaw Dumevi, Joyce Junior Asiamah, Hugette Naa Ayeley Aryee and Dorcas Akuokor Teiko analysed, interpreted/visualized the data. Christopher Yaw Dumevi, Joyce Junior Asiamah, Hugette Naa Ayeley Aryee, Dorcas Akuokor Teiko, Gloria Amoo Aidoo and Gifty Selorm Amenya drafted the manuscript. Christopher Yaw Dumevi, Isaac Ekow Ennin, Eric Essandoh Amuah, James‐Paul Kretchy, Saviour Kweku Adjenti and Patrick F. Ayeh‐Kumi helped to revise the manuscript. All authors contributed equally, read and approved the final manuscript.

## Funding

The authors have nothing to report.

## Ethics Statement

Ethics approval was obtained from the Institutional Review Board of Central University, Accra (Protocol No. CUIRB/02/09/25). The study was conducted in accordance with the Declaration of Helsinki. All data were anonymized and stored in password‐protected files.

## Consent

All study participants provided informed consent prior to data collection. For literate participants, written informed consent was obtained. For participants unable to read, the consent form was read aloud in English and interpreted into the local language (Dangme) in the presence of a witness, and verbal consent was documented with a thumbprint.

## Conflicts of Interest

The authors declare no conflicts of interest.

## Supporting information




**Supporting File 1**: Assessing the Knowledge, Attitude, and Practices (KAP) Regarding Cholera Among Residents of ADA, a Coastal Community in the Greater Accra Region.

**S2 Table 6**: Psychometric Evaluation of the Practices Scale.

**S3 Table 8**: Reliability of extracted factors.

## Data Availability

All relevant data are within the manuscript and its Supporting Information files. The dataset, survey instrument and analysis code supporting this study have been deposited in the Zenodo repository and are publicly available. The persistent digital identifier (DOI) is https://doi.org/10.5281/zenodo.18061946.
