# Supplementary material for: High Awareness, Poor Practice: The Cholera Prevention Knowledge‐Attitude‐Practice Gap in Ada Foah, Ghana—A Cross‐Sectional Study
Source: Public Health Chall. 2026 Aug 13;5(3):e70339. doi: 10.1002/puh2.70339 (PMC13472063; doi:10.1002/puh2.70339)
Supplement: Supplementary file 1 [file PUH2-5-e70339-s001.pdf]

S2 Table 6. Psychometric Evaluation of the Practices Scale

	Scale Mean if Item Deleted	Scale Variance if Item Deleted	Corrected Item-Total Correlation	Squared Multiple Correlation	Cronbach's Alpha if Item Deleted
Do you wash hands with soap after using the toilet?	18.9925	5.220	.381	.563	.111
Do you wash hands before eating/preparing food?	19.0100	5.343	.329	.630	.132
Have you eaten potentially contaminated food?	20.8150	5.815	-.073	.099	.311
Do you cover food to prevent flies?	19.0975	5.391	.210	.555	.162
Do you avoid unwashed fruits/vegetables?	19.3175	5.155	.207	.196	.150
Do you eat street food prepared in unhygienic conditions?	19.9925	6.489	-.210	.203	.365
Do you store drinking water in a covered container?	19.2925	5.466	.085	.105	.213
Do you practice open defecation?	20.1825	6.736	-.268	.201	.380
Do you buy food from hygienic sources?	19.0550	5.691	.107	.228	.206
Have you attended any cholera health education session?	21.1450	5.282	.237	.161	.147
Are you aware of cholera vaccines?	21.0400	5.432	.113	.140	.199
Would you take a cholera vaccine if available?	20.8700	5.407	.210	.119	.163
