# Supplementary material for: High Awareness, Poor Practice: The Cholera Prevention Knowledge‐Attitude‐Practice Gap in Ada Foah, Ghana—A Cross‐Sectional Study
Source: Public Health Chall. 2026 Aug 13;5(3):e70339. doi: 10.1002/puh2.70339 (PMC13472063; doi:10.1002/puh2.70339)
Supplement: Supplementary file 2 [file PUH2-5-e70339-s002.pdf]

S1: QUESTIONNAIRE

ASSESSING THE KNOWLEDGE, ATTITUDE, AND PRACTICES (KAP) REGARDING CHOLERA AMONG RESIDENTS OF ADA, A COASTAL COMMUNITY IN THE GREATER ACCRA REGION.

SOCIO-DEMOGRAPHICS

All responses are confidential and for research purposes only

1. Gender: Male [ ] Female [ ]

2. Age (years):..................................

3. Marital status: Single [ ] Married [ ] Divorced [ ] Widowed [ ] Cohabiting [ ]

4. Religion: Christian [ ] Muslim [ ] Traditionalist [ ] Other: ....

5. Residential area: Urban [ ] Peri-urban [ ] Rural [ ]

6. Highest educational attainment: No formal education [ ] Basic school [ ] Secondary [ ] Tertiary [ ]

7.Primary occupation (*Select one*):

Fishing [ ] Farmer [ ] Government employee [ ] Private sector employee (non-fishing) [ ] Petty trader/Street vendor [ ] Healthcare worker [ ] Teacher/Educator [ ] Student [ ] Unemployed [ ] Other:...

8. Household's primary drinking water source (*Select one*):

Piped water (into home) [ ] Public tap/standpipe [ ] Borehole/well [ ]

Rainwater [ ] Vendor water (tanker) [ ] Surface water (river, lake, pond) [ ]

9. Do you treat water before drinking? Always [ ] Sometimes [ ] Never [ ]

10. Main source of food (*Select one*):

Self-prepared/home-cooked [ ] Street vendors/hawkers [ ] Restaurants/chop bars [ ]

Shared meals with neighbours [ ] Other:..................................

11. Main source of fruits/vegetables (*Select one*):

Homegrown [ ] Local market [ ] Street vendors [ ] Supermarket [ ]

Directly from farmers [ ] Neighbours/friends [ ] 2

KNOWLEDGE

1. Have you heard of cholera? Yes [ ] No [ ] Unsure [ ]

2. How did you hear about cholera? (*Select all that apply*):

Friends/family [ ] Social media [ ] Television [ ] Healthcare workers [ ] NCCE [ ] Other:................................

3. What causes cholera? (*Select all that apply*):

Bacteria [ ] Virus [ ] Parasite [ ] Fungi [ ] Spiritual [ ] Unsure [ ]

4. Possible ways to contract cholera (*Select all that apply*):

Drinking untreated/contaminated water [ ] Eating contaminated food [ ] Poor hand hygiene [ ]

Open defecation near water [ ] Sharing utensils with infected person [ ] Unhygienic food handling [ ]

Raw/improperly prepared seafood [ ] Swimming/fishing in contaminated water [ ] Flies/insects [ ] Breathing air near sick person [ ] Unsure [ ]

5. Symptoms of cholera (*Select all that apply*):

Watery diarrhea [ ] Bloody stools [ ] Vomiting [ ] High fever [ ] Severe dehydration [ ] Muscle cramps [ ] Headache [ ] Unsure [ ]

6. Can cholera be prevented? Yes [ ] No [ ] Unsure [ ]

7. Does poor personal hygiene increase cholera risk? Yes [ ] No [ ] Unsure [ ]

8. Is cholera treatable? Yes [ ] No [ ] Unsure [ ]

9. Can a person get cholera again after recovery? Yes [ ] No [ ] Unsure [ ]

10. Does eating/drinking contaminated food/water increase cholera risk? Yes [ ] No [ ] Unsure [ ]

11. Do infected individuals always show symptoms? Yes [ ] No [ ] Unsure [ ]

12. Does handwashing with soap prevent cholera? Yes [ ] No [ ] Unsure [ ] 3

ATTITUDE

1. Is cholera a serious concern in your community? Yes [ ] No [ ] Unsure [ ]

2. Can cholera lead to death? Yes [ ] No [ ] Unsure [ ]

3. Are you willing to take steps to protect your family from cholera? Yes [ ] No [ ] Unsure [ ]

4. Does washing vegetables reduce cholera risk? Yes [ ] No [ ] Unsure [ ]

5. Do you feel uncomfortable reminding others to wash hands? Yes [ ] No [ ] Unsure [ ]

6. Is personal hygiene critical for cholera prevention? Yes [ ] No [ ] Unsure [ ]

7. Does proper faecal disposal prevent cholera? Yes [ ] No [ ] Unsure [ ]

8. Is seeking medical care important for diarrhea/vomiting? Yes [ ] No [ ] Unsure [ ]

9. Is buying food from dirty vendors risky? Yes [ ] No [ ] Unsure [ ]

PRACTICE

1. Do you wash hands with soap after using the toilet? Always [ ] Sometimes [ ] Never [ ]

2. Do you wash hands before eating/preparing food? Always [ ] Sometimes [ ] Never [ ]

3. Have you eaten potentially contaminated food? Yes [ ] No [ ] Unsure [ ]

4. Do you cover food to prevent flies? Always [ ] Sometimes [ ] Never [ ]

5. Do you avoid unwashed fruits/vegetables? Always [ ] Sometimes [ ] Never [ ]

6. Do you eat street food prepared in unhygienic conditions? Always [ ] Sometimes [ ] Never [ ]

7. Do you store drinking water in a covered container? Always [ ] Sometimes [ ] Never [ ]

8. Do you practice open defecation? Always [ ] Sometimes [ ] Never [ ]

9. Do you buy food from hygienic sources? Always [ ] Sometimes [ ] Never [ ]

10. Have you attended any cholera health education session? Yes [ ] No [ ] Unsure [ ]

12. Are you aware of cholera vaccines? Yes [ ] No [ ] Unsure [ ]

13. Would you take a cholera vaccine if available? Yes [ ] No [ ] Unsure [ ]
