# Supplementary material for: High Awareness, Poor Practice: The Cholera Prevention Knowledge‐Attitude‐Practice Gap in Ada Foah, Ghana—A Cross‐Sectional Study
Source: Public Health Chall. 2026 Aug 13;5(3):e70339. doi: 10.1002/puh2.70339 (PMC13472063; doi:10.1002/puh2.70339)
Supplement: Supplementary file 3 [file PUH2-5-e70339-s003.pdf]

**S3 Table 8. Reliability of extracted factors**

| Scale | Mean ± SD | Cronbach Alpha |
|---|---|---|
| Core Hygiene and Food Protection | 10.69 ± 1.60 | 0.799 |
| Proactive Food and Water Safety | 4.81 ± 1.07 | 0.913 |
| Health System Engagement | 1.24 ± 1.02 | 0.754 |
| Risk Recognition and Vaccine Intent | 1.74 ± 1.09 | 0.862 |
